# Woakes' Syndrome: A Systematic Review of Reported Cases

**DOI:** 10.7759/cureus.99352

**Published:** 2025-12-16

**Authors:** Badrun Shurovi, Benjamin Samra

**Affiliations:** 1 Otolaryngology, Royal London Hospital, London, GBR; 2 General Surgery, Queen Elizabeth Hospital Birmingham, Birmingham, GBR

**Keywords:** crswnp, nasal deformity, nasal polyps, systematic review, woakes' syndrome

## Abstract

Woakes' syndrome is a rare form of chronic rhinosinusitis with nasal polyps (CRSwNP) characterized by the progressive expansion of the nasal framework. We aimed to systematically review the literature on Woakes' syndrome to characterize its clinical features, comorbidities, management strategies, and outcomes.

A literature search was conducted in accordance with the Preferred Reporting Items for Systematic Reviews and Meta-Analyses (PRISMA) guidelines. A systematic search of several databases (PubMed, Embase, Cochrane Library, etc.) was conducted for all reports of Woakes' syndrome. All study types (case reports, case series) were included if they provided clinical data on Woakes' syndrome. Non-English-language papers were excluded. Data on patient demographics, comorbidities (e.g., asthma, aspirin-exacerbated respiratory disease), treatments, and outcomes were extracted and synthesized descriptively.

Twenty-three studies met the inclusion criteria, comprising 39 unique patients (mean age 39.5 years; range 5-81; 65% male). Both paediatric and adult-onset cases were identified. Common comorbidities included Samter's triad (seven cases) and bronchiectasis or sinobronchial disease (two cases). All patients underwent surgical treatment, most commonly functional endoscopic sinus surgery, with adjunctive procedures including digital nasal bone compression (six cases) and formal rhinoplasty or septorhinoplasty (seven cases). Pre- and postoperative steroid therapy was variably reported with most prescribed topical or systemic corticosteroids. Follow-up data were inconsistently reported; where available, the mean follow-up was approximately 12.5 months, with seven documented recurrences (six despite nasal steroid therapy).

Woakes' syndrome is an extremely rare but distinct clinical entity in rhinology, representing an aggressive phenotype of CRSwNP that causes facial deformity. Both paediatric and adult-onset cases occur, frequently associated with underlying conditions such as aspirin-exacerbated respiratory disease in adults. Management requires a combination of aggressive surgical polyp removal and prolonged treatment with steroids to maintain remission. Long-term disease control remains challenging, and emerging treatments such as biological therapies to target chronic inflammation may provide benefit in the most refractory cases.

## Introduction and background

Woakes' syndrome is a rare condition defined by severe, recurrent nasal polyposis with consequent deformity of the nasal pyramid [1]. The origin of the eponym is traced to Edward Woakes, who presented his paper "Necrosing ethmoiditis; its relationship to the development of nasal polypus, Ozæna, &c" [2] in 1885. However, at the time, his theory on the origin of necrotizing ethmoiditis was widely discredited. It wasn't until much later, particularly in French literature, that the condition of nasal polyps with nasal deformity was given the name Woakes' syndrome [3]. In 1979, Kellerhals and de Uthemann published a review further outlining the cardinal features of Woakes' syndrome, including severe recurrent nasal polyps in early childhood with broadening of the nose, nasal dyscrinia (production of abnormally viscous nasal secretions), frontal sinus aplasia, and bronchiectasis [1]. In essence, Woakes' syndrome represents an extreme manifestation of chronic rhinosinusitis with nasal polyps (CRSwNP) that leads to bony erosion and facial disfigurement.

Notably, many patients have comorbid aspirin-exacerbated respiratory disease, and the combination of this with asthma and nasal polyps is known as Samter's triad [4-6]. In contrast, some paediatric cases have been associated with cystic fibrosis or chronic sinobronchial syndromes [7]. Given the potential for disfigurement and complications (such as orbital involvement or even vision loss from sinus expansion), early recognition and intervention in Woakes' syndrome cases are critical [8,9]. In this systematic review, we collate all published cases of Woakes' syndrome, examining their clinical presentations, associated conditions, management strategies, and outcomes in the context of the current understanding of this rare disease.

## Review

Methods

This review was conducted according to the Preferred Reporting Items for Systematic Reviews and Meta-Analyses (PRISMA) guidelines [10]. We performed a comprehensive literature search to identify all publications relating to Woakes' syndrome.

Databases searched included PubMed/MEDLINE, Embase, and the Cochrane Library. The search was performed on October 17, 2025, with the following search terms: for PubMed, "woakes"[All Fields] OR (("nasal polyps/pathology"[MeSH Terms] OR "nasal polyps/physiopathology"[MeSH Terms] OR "nasal polyps/surgery"[MeSH Terms]) AND "nose deformities, acquired/surgery"[MeSH Terms]); for Embase, (('sinonasal polyp'/de OR 'sinonasal polyp') AND ('bone disease'/de OR 'bone disease') OR ('woakes') AND ([embase]/lim OR [medline]/lim OR 'clinical trial':dtype OR [pubmed-not-medline]/lim); and for the Cochrane Library, Woakes OR (MeSH descriptor: [Nasal Polyps] explode all trees AND (MeSH descriptor: [Nose Deformities, Acquired] explode all trees OR nasal deformity OR nasal bone expansion)).

Titles and abstracts were screened by two independent reviewers (BS and BS). Full texts were then retrieved and screened. Inclusion criteria were any case report or case series reporting nasal polyposis with nasal deformity, any age range, and any publication date. Exclusions included non-English texts and summary reviews without patient data.

**Figure 1:**
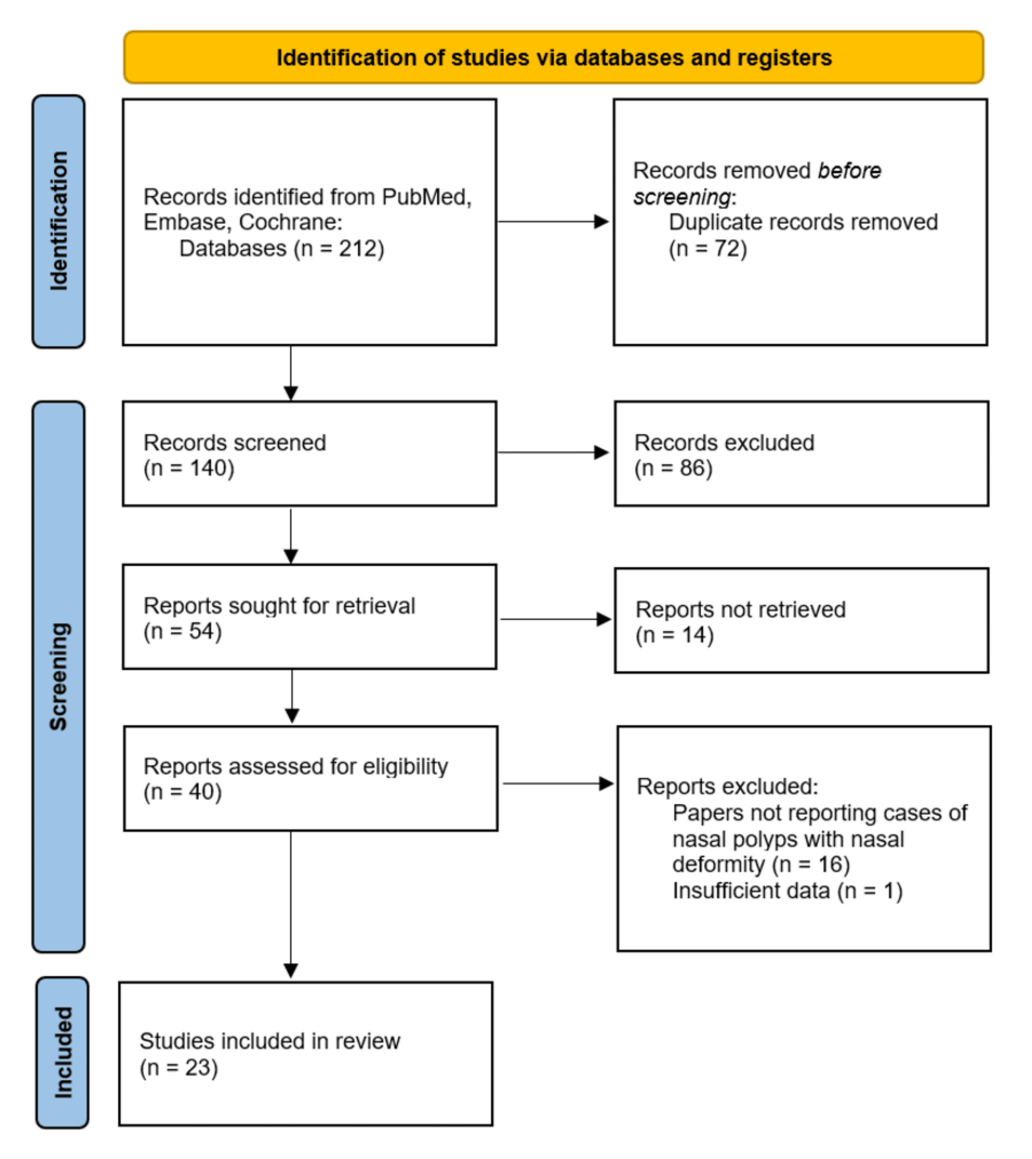
PRISMA flowchart for the study selection PRISMA: Preferred Reporting Items for Systematic Reviews and Meta-Analyses

A PRISMA flow diagram illustrating the study identification and selection process is shown in Figure 1.

Data were extracted by two reviewers from each included study on the following: patient demographics (age, sex), clinical presentation, relevant comorbidities (especially asthma, aspirin-exacerbated respiratory disease, cystic fibrosis, or other immune disorders), any familial occurrence, details of management (surgical interventions and medical therapies), and outcomes (reported recurrence and subjective patient symptoms scores). Given the qualitative nature of the data, results are reported utilizing descriptive statistics.

Results

Study Selection and Characteristics

After removing duplicates and non-relevant papers, we identified 23 publications that met our inclusion criteria, comprising 16 single-patient case reports and seven case series. The largest series, by Günebakan et al., included seven patients treated at a single centre [11]. It should be noted that seven papers of older reports of Woakes' syndrome from the mid-20th century (published in languages such as French and German) were identified during the search but excluded due to a lack of English translation and full texts. For the included studies, publication dates ranged from 1987 to 2025, with a majority in the last decade. In total, 39 unique patients were extracted from the included literature. Table 1 provides a summary of each of the studies and the number of cases presented.

**Table 1:** Summary of the included studies

Study	Number of patients	Patient age (years)	Patient gender	Follow-up length (months)
Abbud-Neme et al. 1987 [12]	1	17	F	36
Aghdam et al. 2022 [9]	1	28	M	NA
Caversaccio et al. 2007 [13]	1	29	M	NA
Chennoufi et al. 2021 [4]	2	55	M	3
		58	F	6
Choi et al. 2019 [14]	1	13	M	NA
Cruz et al. 2019 [8]	2	61	M	NA
		38	M	NA
da Cunha et al. 2014 [15]	1	75	M	16
De Loof et al. 2016 [16]	1	20	M	12
Dickie et al. 2022 [5]	3	42	M	4
		49	F	6
		51	M	12
El Fakiri et al. 2019 [17]	1	42	M	6
Groman et al. 2004 [7]	2	21	F	NA
		18	F	NA
Gunebakan et al. 2022 [11]	7	19	M	24
		48	M	32
		51	F	18
		35	M	12
		41	M	16
		42	M	22
		29	F	24
Hazwan et al. 2019 [18]	1	44	M	12
Holgado et al. 2025 [19]	1	71	M	NA
Kellerhals and de Uthemann 1979 [1]	4	6	F	12
		15	M	12
		5	M	12
		5	F	12
Lapusneanu et al. 2021 [20]	1	60	M	3
Salvador et al. 2021 [21]	1	68	F	12
Schoenenberger and Tasman 2015 [22]	1	29	F	8
Tajima et al. 2023 [23]	1	66	M	NA
Ueda et al. 2017 [24]	1	55	F	6
Wardani and Mayangsari 2014 [25]	1	16	M	4
Winestock et al. 1978 [26]	4	63	F	3
		18	F	7
		81	M	NA
		40	M	NA

Patient Demographics

Patient ages ranged from five to 81 years. Seven patients were younger than 18 years at presentation, and 33 patients were adults, with a mean reported age of 39.5 years. The sex distribution was predominantly male with a male-to-female ratio of 1.86:1 (summarized in Table 2).

**Table 2:** Summary of patients' demographics

Category	Number of patients (%)
Paediatric (<18)	7 (18%)
Adult (≥18)	32 (82%)
Male	25 (64%)
Female	14 (36%)
Mean age	39 years
Age range	5-81 years

The distribution of reported comorbidities is as follows: seven patients had Samter's triad (asthma and aspirin-exacerbated respiratory disease); three had aspirin intolerance reported without asthma; two patients had bronchiectasis or sinobronchial syndrome; one patient had oculocutaneous albinism; one patient had eosinophilic otitis media; two patients were siblings with familial nasal polyposis; and three patients developed orbital complications.

Medical Therapy

Preoperatively, most studies did not specify any medical therapy. In cases where preoperative medical therapy is reported, eight cases were treated with a combination of oral and nasal steroids. Oral steroids were used as monotherapy in seven cases, nasal steroids were used as monotherapy in two cases, and a further two cases were treated with intravenous steroids. Oral antibiotics were also prescribed in seven cases preoperatively.

Postoperatively, the most common treatment was with nasal steroids alone, reported in 14 cases. Oral steroids were used in six cases, and combined topical and oral steroids were used in two cases. Only one case reported intravenous steroid usage. Antibiotics were prescribed in seven cases. A single case report also described the use of radiotherapy postoperatively, despite the histology confirming benign disease.

Surgical Management

The mainstay of treatment for all patients was polypectomy, with a variety of other procedures performed depending on the indication. Four patients underwent maxillary sinus fenestration, and four patients had ethmoidectomy. A further four patients had more extensive surgeries, with two having sphenoethmoidectomy, one requiring fronto-spheno-ethmoidectomy, and the final patient having middle turbinate resection. Additionally, two patients required orbital decompression from the pressure effects of the polyps.

In terms of reconstructive techniques, the most commonly reported procedure was external digital compression (in which pressure is applied to the nasal bridge during general anaesthesia to achieve the desired cosmetic result), which was reported in six cases. Formal septorhinoplasty was undertaken in five patients, and rhinoplasty with osteotomy was required in two patients.

Histology

Only 25 cases reported the histology of the polyp specimens resected. Of these, five reported eosinophilic infiltrates. 

Outcomes

The overall reporting on follow-up was poor, with no follow-up reported in 11 (27.5%) cases. The average length of follow-up reported was 12.5 months, with seven cases reporting recurrence. In six of these cases, the polyps recurred despite steroid therapy. All cases reported initially positive postoperative outcomes with patient reports of improved symptoms and aesthetics. Only one study quantified these via the Sino-Nasal Outcome Test-22 (SNOT-22) [17], which is a clinical quality of life questionnaire. In this study, at six months, the patient's score reduced from 72/110 to 16/110.

Discussion

Woakes' syndrome has historically been described as a condition of childhood [1]. However, the present review demonstrates that most published cases occur in adults. This distribution likely reflects a reporting bias rather than a true epidemiological trend, as paediatric cases may be underreported. Adult-onset Woakes' syndrome may also represent the extreme end of the CRSwNP spectrum, manifesting only after years of progressive disease.

Comorbidities in reported cases support a possible but not definitive association with asthma and aspirin sensitivity. Several patients met the criteria for Samter's triad [6], which suggests that while aspirin-exacerbated respiratory disease may act as a contributing factor in some cases, it is not an essential feature of Woakes' syndrome. Nonetheless, the pathophysiological overlap with CRSwNP implies a shared inflammatory basis. Previous work has identified that type 2 inflammation via IL-4 and IL-5 [27] is the primary driver of CRSwNP and eosinophilic infiltrates have been shown to have a major role and target for therapy [28]. Currently, there are international recommendations for biological therapies for patients with bilateral polyps after functional endoscopic sinus surgery [29]. Within the cases presented, there was limited examination of the histopathology, with only five cases reporting extensive eosinophilic infiltrates. Therefore, future work will need to examine the pathology of Woakes' cases to determine if the pathophysiology is the same as CRSwNP or represents a different, more inflammatory pathway.

Medical management alone, particularly with oral or topical corticosteroids, appears to offer limited efficacy in controlling symptoms or preventing progression in these patients. This is in marked distinction to the majority of CRSwNP, where nasal steroids have been proven to be effective at reducing polyp size as well as the risk of recurrence [29]. Therefore, surgical intervention remains the cornerstone of treatment. Functional endoscopic sinus surgery, tailored to the extent of disease, allows the effective clearance of polyps and the restoration of sinus ventilation. In cases with significant external nasal deformity, concurrent reconstructive surgery (in the form of external digital compression) has been consistently reported as safe and effective, achieving both functional and aesthetic improvement without increased complication rates [4,16,22].

One outlier case employed postoperative radiotherapy despite benign histology, with the subsequent recurrence of polyps during follow-up. This isolated experience, coupled with the benign inflammatory nature of the disease, indicates that radiotherapy is inappropriate and ineffective for Woakes' syndrome. Although no biologic agents were used in the cases reviewed, their potential role in refractory or recurrent polyp disease warrants consideration in the future management of this condition.

Several limitations must be acknowledged. All included studies were case reports or small series, inherently limiting the strength and generalizability of the conclusions. There is a high likelihood of publication bias, as severe or atypical presentations are more likely to be documented. Consequently, the true spectrum of disease severity remains unclear, and quantitative synthesis was not feasible. Only English-language publications were included, primarily due to the inaccessibility of non-English full texts. This exclusion omits several early 20th-century reports, particularly in French, which historically described the majority of cases. However, the clinical and surgical relevance of these older cases is uncertain given advances in surgical and medical management.

## Conclusions

Woakes' syndrome remains a rare but important clinical entity in rhinology and otolaryngology. This systematic review has consolidated the findings from the limited literature available. Woakes' syndrome appears to present in two peaks, childhood and adulthood, and is frequently associated with systemic inflammatory comorbidities, notably aspirin-exacerbated respiratory disease and asthma. Management of the condition requires a combined multidisciplinary approach: surgical removal of the polyps (via functional endoscopic sinus surgery and when feasible), simultaneous correction of the nasal deformity with external digital compression, and long-term medical therapy to control inflammation. The reported short-term outcomes for all patients were positive. However, in the longer follow-up, there were a significant number of recurrences, which highlights the need for ongoing observation/follow-up of patients. Clinicians should be aware of Woakes' syndrome and consider it in any patient with extensive nasal polyps and a broadening nasal bridge. While current treatments can effectively address the disease, the rarity of Woakes' syndrome means that each case adds valuable information. Hence, ongoing documentation of cases, as well as research into underlying immune or genetic factors, will be essential to fully understand Woakes' syndrome and to identify potential future therapies.
